# A Simple and Fast Kinetic Assay for the Determination of Fructan Exohydrolase Activity in Perennial Ryegrass (*Lolium perenne* L.)

**DOI:** 10.3389/fpls.2015.01154

**Published:** 2015-12-22

**Authors:** Anna Gasperl, Annette Morvan-Bertrand, Marie-Pascale Prud’homme, Eric van der Graaff, Thomas Roitsch

**Affiliations:** ^1^Institute of Plant Sciences, Karl-Franzens-Universität GrazGraz, Austria; ^2^Normandie Université, CaenFrance; ^3^UMR 950 Ecophysiologie Végétale, Agronomie et Nutritions NCS, Université de Caen NormandieCaen, France; ^4^INRA, UMR 950 Ecophysiologie Végétale, Agronomie et Nutritions NCSCaen, France; ^5^Department of Plant and Environmental Sciences, Copenhagen Plant Science Centre, University of CopenhagenCopenhagen, Denmark

**Keywords:** 1-FEH, enzymatic activity, fructan exohydrolase, fructan degradation, kinetic assay, perennial ryegrass

## Abstract

Despite the fact that fructans are the main constituent of water-soluble carbohydrates in forage grasses and cereal crops of temperate climates, little knowledge is available on the regulation of the enzymes involved in fructan metabolism. The analysis of enzyme activities involved in this process has been hampered by the low affinity of the fructan enzymes for sucrose and fructans used as fructosyl donor. Further, the analysis of fructan composition and enzyme activities is restricted to specialized labs with access to suited HPLC equipment and appropriate fructan standards. The degradation of fructan polymers with high degree of polymerization (DP) by fructan exohydrolases (FEHs) to fructosyloligomers is important to liberate energy in the form of fructan, but also under conditions where the generation of low DP polymers is required. Based on published protocols employing enzyme coupled endpoint reactions in single cuvettes, we developed a simple and fast kinetic 1-FEH assay. This assay can be performed in multi-well plate format using plate readers to determine the activity of 1-FEH against 1-kestotriose, resulting in a significant time reduction. Kinetic assays allow an optimal and more precise determination of enzyme activities compared to endpoint assays, and enable to check the quality of any reaction with respect to linearity of the assay. The enzyme coupled kinetic 1-FEH assay was validated in a case study showing the expected increase in 1-FEH activity during cold treatment. This assay is cost effective and could be performed by any lab with access to a plate reader suited for kinetic measurements and readings at 340 nm, and is highly suited to assess temporal changes and relative differences in 1-FEH activities. Thus, this enzyme coupled kinetic 1-FEH assay is of high importance both to the field of basic fructan research and plant breeding.

## Introduction

Water-soluble carbohydrates are stored either as starch or fructans (Hendry, 1993). Starch metabolism and the associated primary carbohydrate metabolism have been studied extensively. The enzymes involved have been characterized in detail, assays developed for determining the associated enzyme activities and biochemical properties, and the corresponding genes encoding these enzymes have been cloned from many different plant species (Bahaji et al., 2014). These enzyme activity assays are often enzyme coupled reactions (Appeldoorn et al., 1997, 1999; Pelleschi et al., 1997; Manjunath et al., 1998; Petreikov et al., 2001; Bisswanger, 2004) and relatively easy to perform. In contrast, the characterization of enzymes involved in fructan metabolism and the genes encoding these enzymes is less advanced.

Fructans were shown to be important for abiotic stress tolerance (Ruuska et al., 2008; Valluru and Van den Ende, 2008; Livingston et al., 2009) and regrowth of leaf tissue after defoliation (Morvan-Bertrand et al., 2001; Lee et al., 2011). Fructans are also linked to dietary benefits (Ritsema and Smeekens, 2003; Vogt et al., 2013; Peshev and Van den Ende, 2014). More recently, fructans have attracted attention as source for the conversion of fructose into the platform molecule 5-Hydroxymethylfurfural (Gallezot, 2012), which serves as one of the most promising platform molecules for future sustainable production of an unequaled wide range of bio-based products including esters, biopolymers, pharmaceuticals, food ingredients, agrochemicals, and biofuel. Despite the importance of fructan metabolism for temperate grasses (Pollock and Cairns, 1991) and monocot staple food crops (Rasmussen et al., 2009), the knowledge on the enzymes involved in fructan metabolism and their corresponding encoding genes is still limited (De Coninck et al., 2007; Van den Ende, 2013). Further, the availability of gene sequences is mostly limited to partial clones, or not available for many of the plant species that (predominantly) accumulate fructans as carbohydrate storage form.

In addition to the synthesis of fructans, involving both *de novo* synthesis using sucrose as the starting point and fructan chain elongation, specific enzymes called fructan exohydrolases (FEHs EC 3.2.1.153) degrade fructan chains. FEH activity liberates the stored fructose (Van den Ende et al., 2003; Lothier et al., 2007) to enable outgrowth of new leaves, promote abiotic stress tolerance (De Coninck et al., 2007) and seed filling (Zhang et al., 2015). FEHs also perform trimming of fructan molecules (Bancal et al., 1992; Hincha et al., 2000; Vereyken et al., 2001; Valluru et al., 2008; Livingston et al., 2009) to generate fructan molecules of specific degree of polymerization (DP), which is for example important to generate low DP fructans for membrane stabilization (Hincha et al., 2000; Vereyken et al., 2001). FEH enzymes differ by the linkage type of fructan (ß-2,1 or ß-2,6) they preferentially hydrolyze. Several types of FEHs have been found in monocots: 1-FEH, 6&1-FEH, and 6-FEH (Marx et al., 1997; Van den Ende et al., 2003; Kawakami et al., 2005; Van Riet et al., 2008). Two of these FEH enzymes have been identified and characterized in perennial ryegrass (*Lolium perenne* L.), 1-FEH and 6-FEH (Marx et al., 1997; Lothier et al., 2007, 2014).

Fructan exohydrolase activity was found to be quickly increased following defoliation in grasses (Marx et al., 1997; Morvan-Bertrand et al., 2001; Tamura et al., 2011). This increase of FEH activity involves transcriptional and/or post-translational regulation, depending on species and FEH isoforms (Lothier et al., 2007, 2014; Tamura et al., 2011), and may be triggered through sugar signaling (Lothier et al., 2010). *FEH* expression was furthermore triggered by cold treatment in a number of fructan accumulating grasses (Kawakami et al., 2005; del Viso et al., 2009; Rao et al., 2011*;* Kawakami and Yoshida, 2012). Hence, analysis of FEH activity is important to understand fructan synthesis and metabolism during plant development and various stress conditions (Zhang et al., 2015).

Assays to determine the activity of enzymes involved in fructan metabolism and to study their biochemical properties require expensive HPLC equipment and specially trained and experienced personal using it. Further, the specific fructan sources that have to be used as HPLC standards and substrates for these assays are often very expensive or even not commercially available. Therefore, fructan enzyme analyses are restricted to specialized labs with access to such equipment and specified fructan sources. Thus, enzyme coupled reactions, such as developed to determine the activities for enzymes involved in primary carbohydrate and starch metabolism (Jammer et al., 2015), are desirable to make fructan enzyme analyses accessible to any lab. This will contribute to improve the characterization of fructan metabolism and the enzymes involved.

Standard measurement of 1-FEH activity is performed using endpoint assays (Lothier et al., 2007), followed by product detection either via HPLC analysis or as recently described via an enzyme coupled assay (Lothier et al., 2010). Based on published enzyme coupled assay protocols for 1-FEH activity performed in single cuvettes (Lothier et al., 2007; Krivorotova and Sereikaite, 2014), we set out to adapt these protocols for use in a 96-well format, similar to the miniaturization of assays for 13 key enzymes of primary carbohydrate metabolism (Jammer et al., 2015). This would enable a high throughput assay allowing the handling of many samples in parallel and saving chemical costs by performing these assays in a small volume. Recently, the enzyme coupled assay for 1-FEH was shown to be superior to two alternative methods employing dyes (Krivorotova and Sereikaite, 2014) indicating the importance of such a simple and fast 1-FEH assay for the fructan community. We show that the 1-FEH enzyme coupled assay was successfully adapted to study 1-FEH activity in perennial ryegrass, which serves as model species to study fructan metabolism (Prud’homme et al., 2007; Lee et al., 2010). In addition we included one case study, showing the expected increase in 1-FEH activity during cold treatment using 1-kestotriose as substrate, thereby validating this 96-well enzyme coupled kinetic 1-FEH assay. Recently, 1-FEH activity was identified as potential important determinant for grain yield because of remobilization of stem WSC, especially under drought conditions (Zhang et al., 2015). Therefore, 1-FEH activity represents an important new target for breeding in agronomic important species such as wheat and barley. Thus, this simple and fast enzyme coupled kinetic 1-FEH activity could be an important tool, also for small and medium size breeders that often do not have access to specialized and sophisticated HPLC equipment, to improve the important traits of drought adaptation and high sugar content.

## Material and Methods

### Plant Material

For generation of plant material for the optimization of the enzyme coupled kinetic 1-FEH assay, perennial ryegrass (*L. perenne* L. genotype Aberchoice) was grown from seeds. Seeds were sown densely in vermiculite (Pull Rhenen B.V., The Netherlands), in 6 cm pots and transferred into a growth cabinet (Pol Eko, Poland), equipped with Master TL5 Ho lamps with 54 W (Phillips, The Netherlands) for germination and growth of the seedlings. Seedlings were grown for 4 weeks under long day conditions: 16 h light and 20°C (day)/18°C (night), respectively, and 70% humidity.

For the case study experiment to assess the impact of cold treatment of FEH activity, perennial ryegrass (*L. perenne* L. genotype Aberchoice) was grown under greenhouse conditions in vermiculite by the cooperation partner Saatzucht Steinach in Steinach, Germany. Plants were transferred to Graz and kept under greenhouse conditions, where additional light at 39.6 μmol s^-1^ m^-2^ was supplied by Plug and Grow^TM^ 154 200 W 6400 K fluorescent lamps (Trade Hydro, UK). To maintain these plants they were cut at 3 cm above ground level every 45 days to achieve dense stubble. Adult, ca. 2 years-old plants were transferred to a growth cabinet HPS 1500/2000 (Vötsch Industrietechnik GmbH, Germany), equipped with Philips bulbs (Philips Austria GmbH, Graz, Austria), which provided 28.6 μmol s^-1^ m^-2^ light intensity at plant level. Plants were fertilized weekly with a 50 g L^-1^ solution of Ferty 9 Hydro (Planta Düngemittel GmbH, Germany). 160 Plants were again grown under long day conditions: 16 h light and 20°C (day)/18°C (night), respectively, and 70% humidity.

### Harvesting and Cold Treatment

After germination seedlings were grown for 4 weeks in the growth cabinets and harvested 4 h after the light was turned on to generate material for the optimization of the enzyme coupled kinetic 1-FEH assay. The growth stage corresponds to the early vegetative growth stages V1/V2 according to the mnemonic growth code of Moore et al. (1991) with the first or second leaf collared. The stubble length was ca. 2 cm, the leaf length 2–3 cm. Seedlings were cut at ground level and again 1 cm above ground level, representing the mixed sink tissue stubble, i.e., the enclosed immature sink leaf laminae and immature and mature sink leaf sheaths. Material of 30 seedlings was pooled for each individual biological sample, immediately frozen in liquid nitrogen and stored at -80°C until further use.

For the case study experiment (cold treatment) ca. 2 years-old, frequently cut back adult plants were used that had formed a dense, clonal patch and that were characterized by 2–4, ca. 12–15 cm long leaves emerging from 3 to 4 cm long sheaths. Prior harvest, the adult plants were cut back at 3 cm above ground level and subsequently grown for 3 weeks. The experiments started by harvesting untreated control samples 4 h after the light was turned on. Plants were cut at ground level and again 3 cm above ground level, representing mixed sink tissue. Material from two plants was pooled for each individual biological sample, immediately frozen in liquid nitrogen and stored at -80°C until further use. The remaining plants were acclimated to short day and cold conditions: 8 h light and 6°C (day)/2°C (night), respectively and 70% humidity. Cold treated samples were harvested after 1, 2, 3, 4, 10, and 14 days, again 4 h after the light was turned on.

Prior to extraction and analyses, frozen plant material was pre-homogenized in liquid nitrogen with mortar and pestle, while 0.1% PVPP (Sigma-Aldrich, Germany) was added to bind phenolic compounds. These mixtures were homogenized to fine powder, in a MM400 ball mill (Retsch, GmbH, Germany). Ground plant material was stored at -80°C until further use.

### Extraction of Proteins and Kinetic 1-FEH Enzyme Activity Coupled Enzyme Assay

Protein extracts were made from frozen ground material as described by Pavis et al. (2001a). After removal of plant material residues via centrifugation, the supernatant was subjected to dialysis according to Jammer et al. (2015) to remove highly abundant inhibitors (i.e., sucrose; Lothier et al., 2007) and reaction products (i.e., fructose). Protein extracts were snap frozen in liquid nitrogen and stored at -20°C in small aliquots until further use.

**FIGURE 1:**
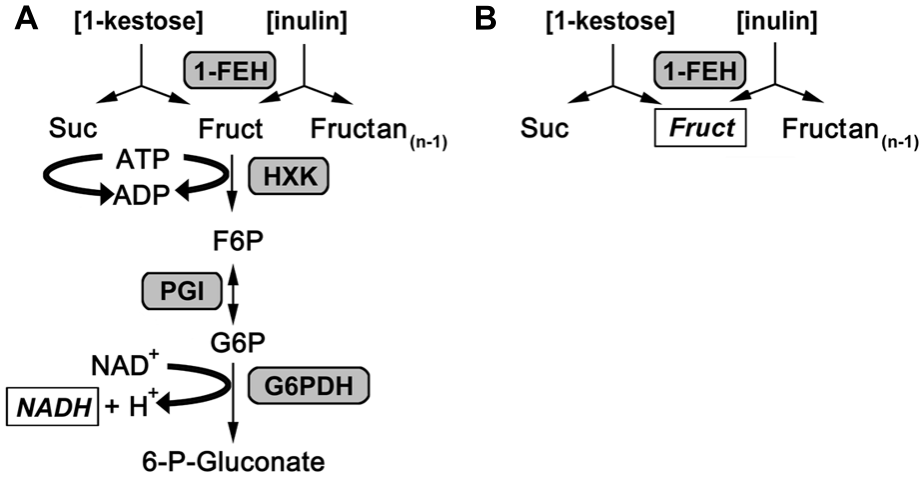
**Reaction scheme for 1-FEH activity assays. (A)** Reaction scheme for the enzyme coupled kinetic 1-FEH activity assay. **(B)** Reaction scheme for endpoint 1-FEH activity. Either 1-kestose (1-kestotriose) or inulin was used as substrate. The products determined to calculate 1-FEH activity are italicized and shown in square boxes.

An endpoint assay for 1-FEH activity of plant samples (Lothier et al., 2007; Krivorotova and Sereikaite, 2014) was adapted for a kinetic approach measuring plant extracts in 96 well-format and monitoring enzymatic activity via an increase in NADH (**Figure 1**), detected by UV/VIS spectroscopy at 340 nm with an Ascent Multiskan microplate reader (Thermo Scientific, USA) as described for the miniaturization of assays to determine carbohydrate enzyme activities by Jammer et al. (2015). For the kinetic 1-FEH activity assay, 10 μL of dialyzed enzyme extract were incubated with 1 mM EDTA set to pH 8 with 10 M NaOH, 2 mM MgCl_2_ (Carl Roth, Germany), 50 mM 1-kestotriose (1-K; Wako Chemicals GmbH, Germany) or 5% inulin from chicory root (Sigma), 1.3 mM ATP (AppliChem, Germany), 0.5 mM NAD, 5 mM DTT (Carl Roth), 0.4 U glucose 6-phosphate dehydrogenase from *Leuconostoc mesenteroides* (G6PDH EC 1.1.1.49, Oriental Yeast, Japan), 0.672 U hexokinase from yeast (HK 2.7.1.1, Roche, Germany) and 0.56 U phosphoglucose isomerase from yeast (PGI 5.3.1.9, Roche), added up to a total assay volume of 160 μL with 80 mM citrate phosphate buffer, pH 5.5. In this enzyme coupled kinetic 1-FEH assay the helper substrates are MgCl_2_, ATP and, NAD, whereas the auxiliary enzymes used are hexokinase (HXK), PGI and glucose-6-dehydrogenase (G6PDH). The 1-FEH enzyme activity is the rate limiting step and detected via increase in NADH at 340 nm, while 1-FEH activity is detected via increase in fructose by HPLC for the standard endpoint assay (**Figure 1**). Measurement of enzymatic activity was performed as described for carbohydrate enzyme activities by Jammer et al. (2015) in 96-well plate readers suited for kinetic assays at 340 nm. Specific enzyme activity was calculated during the linear phase of substrate conversion, corrected for background from blanks or controls and expressed in nkat.gFW^-1^. Invertase enzyme activities were determined as described before (Jammer et al., 2015), while expression analysis for *Lp1-FEHa* and *Lp1-FEHb* was performed as described in Gasperl et al. (submitted: accompanying paper submitted to FiPS).

### Statistical Analyses

Results were expressed as the mean of three biological replicates + SE. Student’s *t*-test in Microsoft Excel 2007 software was used for statistical analysis.

## Results and Discussion

### Optimization of the Kinetic 1-FEH Assay

1-FEH activity was determined in this assay via coupled enzyme activities as previously described (Lothier et al., 2007; Krivorotova and Sereikaite, 2014). Fructose was released from the substrate (inulin or 1-kestotriose) via plant 1-FEH activity and subsequently phosphorylated to fructose 6-phosphate via hexokinase, isomerized to glucose 6-phosphate by PGI and oxidized by NAD^+^ through glucose 6-phosphate dehydrogenase (**Figure 1**). The increase in NADH as measure for 1-FEH activity was monitored at 340 nm for 20 min. Since the auxiliary enzymes are provided in excess, the rate limiting step is the 1-FEH activity and its product fructose is directly converted to 6-phosphogluconate. This prevents other enzymes in the protein extracts to act on fructose, which is an advantage of this kinetic activity assay over the standard HPLC endpoint assay for 1-FEH. To correct for background activity from plant extract, or substrate, blank measurements (omitting substrate) and controls (omitting plant extract) were analyzed in parallel to the plant samples. All measurements were performed in 96-well-format microtiter plates, which enabled us to run 12 biological samples (60 reactions) in parallel with the actual 1-FEH activity measurement as three technical replicates, one blank and one control reaction per sample within 20 min run time. Thereby, our kinetic assay proved to be time saving compared to endpoint assays that usually take several hours (Morvan et al., 1997) to which the time required for HPLC analysis of the end products has to be added.

A special advantage of kinetic enzyme assays over endpoint measurements is that the enzymatic activity over time is followed in each measurement independently. Typically, the linear phase of the enzyme coupled kinetic assays for 1-FEH (this study) and carbohydrate enzyme activities (Jammer et al., 2015) were reached after a variable lag phase. Sometimes saturation of the reaction was reached toward the end of a measurement, depending on the biological sample and/or extract amount. To ensure the optimal and precise determination of enzyme activities, each activity plot was manually inspected to specify the linear phase for calculation of the enzyme activities. Further, this made it possible to check the quality of any reaction with respect to linearity of the kinetic assays, providing the possibility to directly identify the eventual presence of negative factors when analyzing novel species, which could be missed in endpoint reactions. Therefore, the enzyme coupled kinetic 1-FEH assays enable an optimal and more precise determination of 1-FEH activities compared to endpoint assays.

**FIGURE 2:**
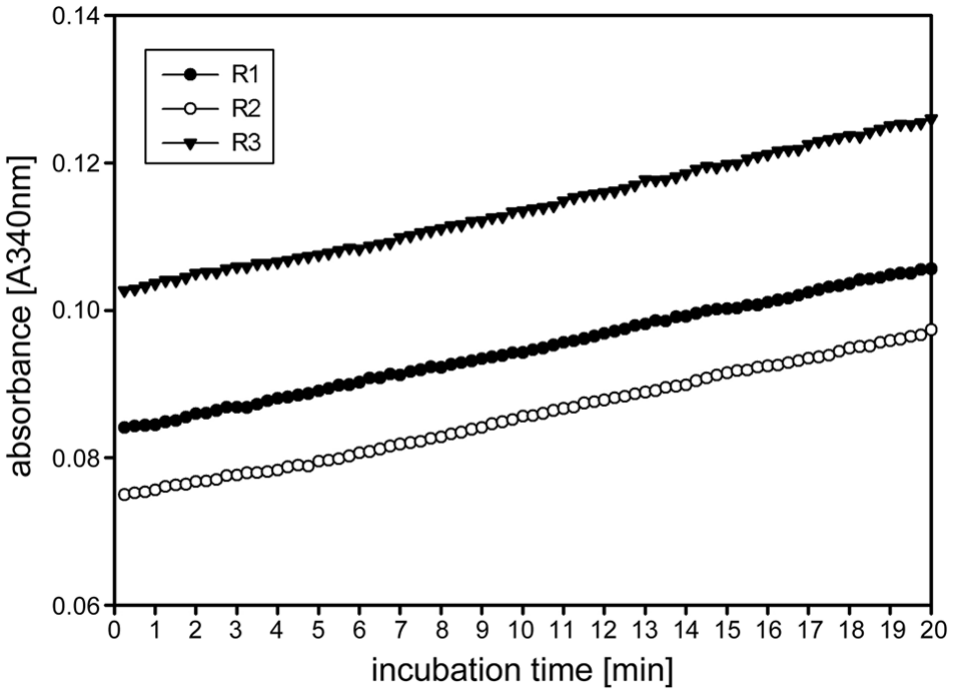
**Time linearity and stability of the kinetic 1-FEH assay.** Time dependent linearity for 1-FEH activity in three independent biological samples (R1-3), each determined as three technical replicates experiments, shown as absorbance [A^340^] over 20 min. 8 μL extract from stubble of 4-weeks-old perennial ryegrass Aberchoice plants. Enzyme activity was analyzed in the first centimeter of stubble from ground level, using 50 mM 1-kestotriose as substrate.

**FIGURE 3:**
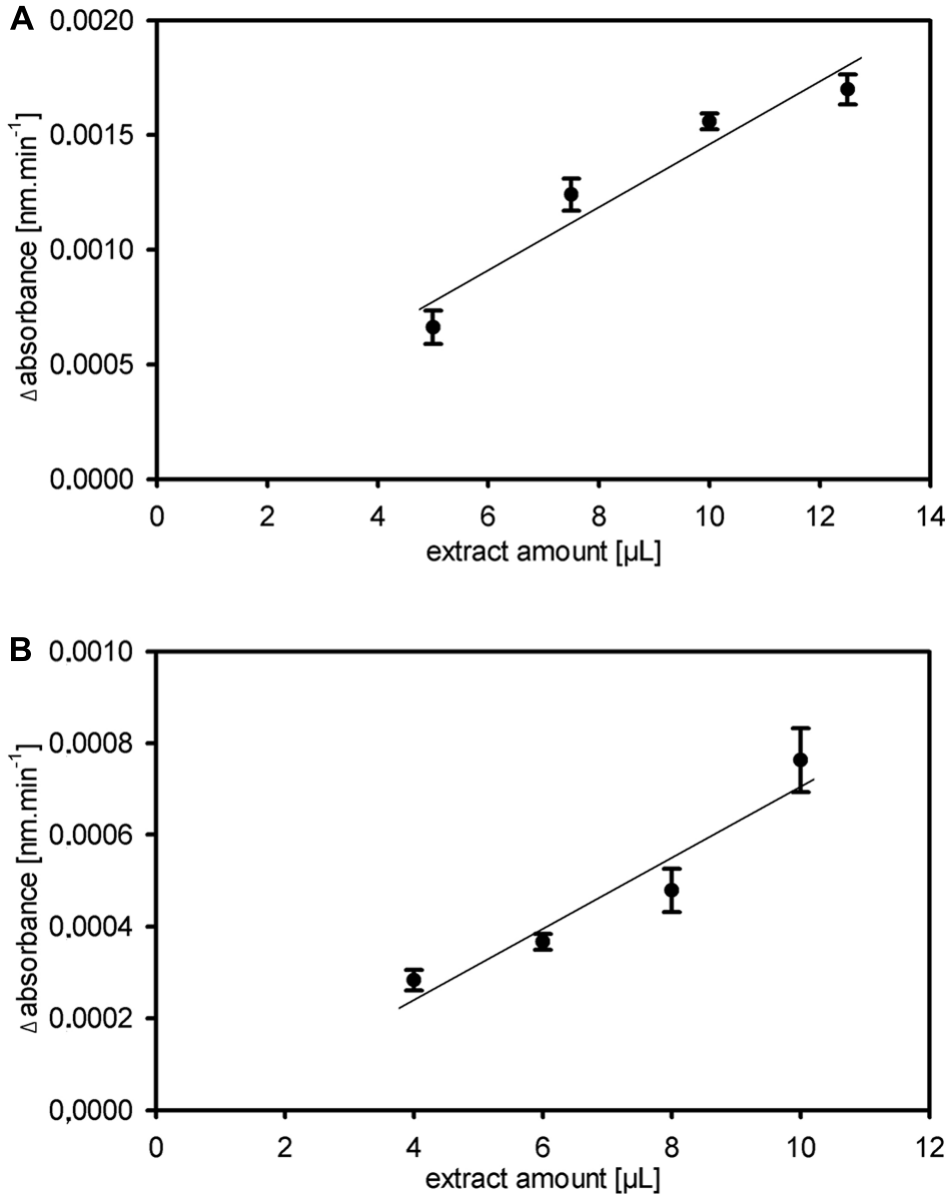
**Robustness and stability of the kinetic 1-FEH assay. (A,B)** Dose dependent linearity for extract amount of fructan 1-FEH activity shown as Δabsorbance [nm.min^-1^], in two independent experiments using stubble of 4-weeks-old perennial ryegrass Aberchoice plants **(A)** Experiment 1 **(B)** Experiment 2. Values in graph **(A)** represent the mean of three technical replicates from the same plant extract + SE; values in graph **(B)** the mean of three biological replicates + SE.

**FIGURE 4:**
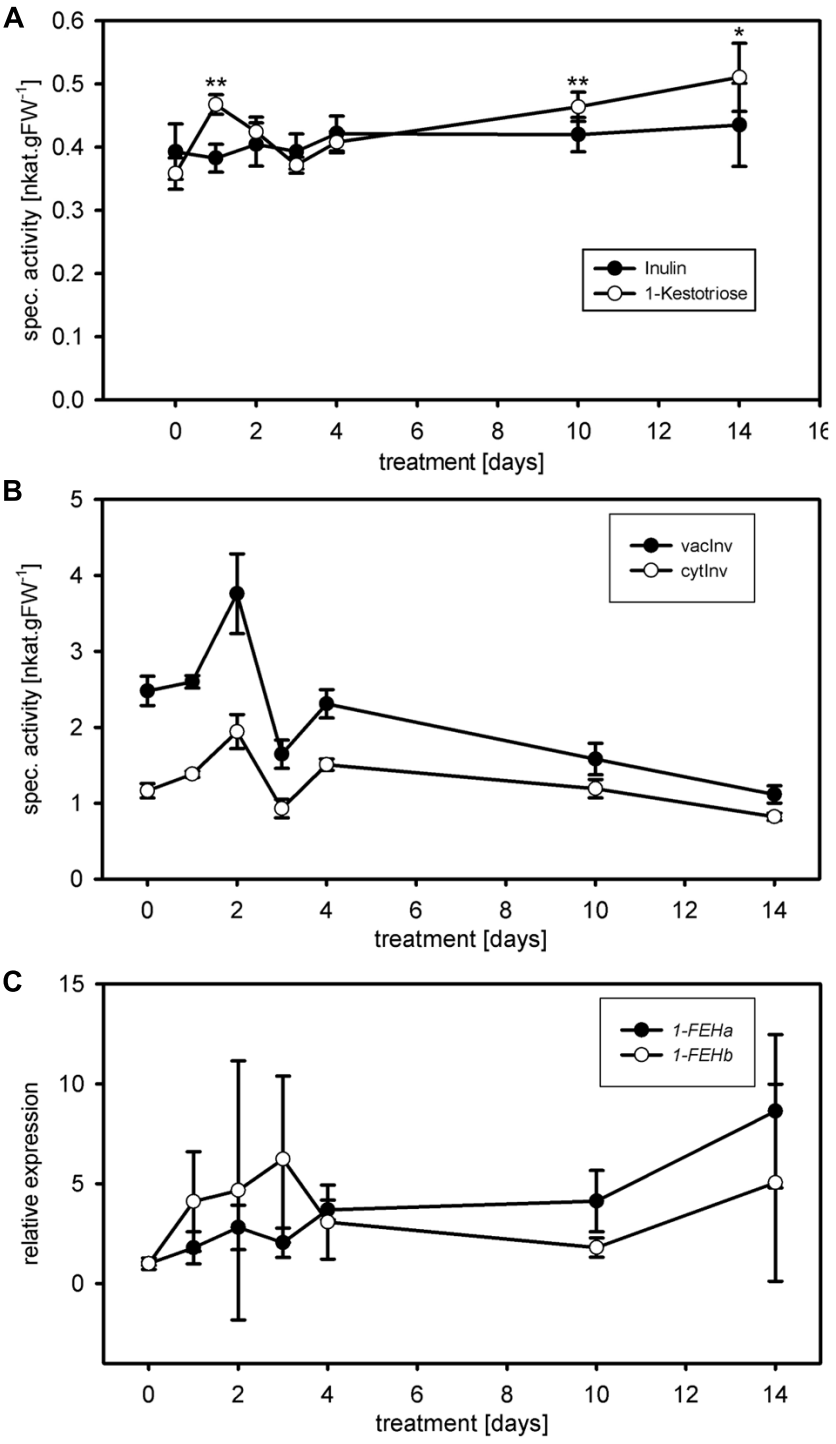
**Case study and substrate comparison for 1-FEH activity. (A)** 1-FEH activity (nkat.gFW^-1^) was determined during cold treatments (case study) using either 50 mM 1-kestotriose (open circles) or 5% inulin (closed circles) as substrate. Under control conditions (*t* = 0), both substrates resulted in similar 1-FEH activities. Following cold treatment (10 and 14 days), 1-FEH activity showed the expected increase using 1-kestotriose as substrate, coinciding with increased *Lp1-FEH* expression levels. No significant change in 1-FEH activity is observed using inulin as a substrate. **(B)** cytInv activity (open circles) and vacInv activity (closed circles) during cold treatment. Both invertase isoenzyme activities showed a continuous decrease from days 4 to 14. **(C)** Quantitative RT-PCR analysis of *Lp1-FEHa* (closed circles); *Lp1-FEHb* (open circles) expression during cold treatments. Transcript levels were normalized to untreated controls (expression at *t* = 0). Values in the graphs represent the mean of three biological replicates ± SE. Asterisks indicate statistical significant differences from control treatment (*t* = 0) at ^∗^*p* < 0.05 and ^∗∗^*p* < 0.01, respectively.

Other enzymes present in the protein extracts used for enzyme activity assays could (1) compete for the same substrate, (2) convert intermediates or the final product or (3) produce the same end product used for detection of the enzyme activity. This could result in over- or underestimation of the studied enzyme activity. Important aspects of kinetic assays are linearity of the reaction in time and stability of the reaction, enabling to calculate the enzyme activity over many data points to ensure high accuracy of the enzyme activities. We routinely obtained linearity of the kinetic assays over the full 20 min run time, occasionally after a short lag phase of several minutes, for our miniaturized 1-FEH kinetic assay (**Figure 2**). Assays for biological replicates showed the reproducibility of the recorded enzyme activities and stability of the reactions (**Figure 2**). Further, it is important to determine the optimal amount of substrate and extract for different plant species and/or organs assessed, to obtain the optimal assay conditions within the linear phase. Since the optimal assay parameters differ for each of the enzymes present in the protein extracts, ‘contaminating’ or ‘competing’ enzyme activities would be obvious from kinetic plots that deviate from linearity. This is best exemplified by the enzyme coupled kinetic sucrose synthase activity assay (Jammer et al., 2015), which is performed as two independent assays, one detecting combined sucrose synthase and invertase activity, and a second assay detecting only the background invertase activity. This background invertase activity is evident in the assay detecting simultaneous sucrose synthase and invertase activity from a biphasic instead of linear activity plot (data not shown). Technical and biological replicates showed that the optimal extract amount ranged from 7.5 to 10 μL for ryegrass stubble from seedlings (**Figure 3**) and mature plants (data not shown). The robustness and stability of the kinetic 1-FEH assay, both for technical and biological replicates, is successfully shown in independent experiments (**Figure 2**–**4**). 1-FEH activity detected via the optimized enzyme coupled kinetic assay (**Figure 4**) is in the same range as previously described for perennial ryegrass samples (Lothier et al., 2007). The linear plots for 1-FEH activity in respect of time and extract amount obtained with the enzyme coupled kinetic 1-FEH assay suggests that the influence from other enzymes in the protein extract was neglectable.

### Substrate Comparison

The use of the adequate substrate is equally important, next to time and dose dependent linearity of enzymatic activity, to ensure robustness and accuracy of the assay. The optimum substrate for ryegrass FEH is high-molecular-weight fructan derived from ryegrass (Morvan et al., 1997) as used by Lothier et al. (2010). However, this extraction and purification procedure requires time and expensive HPLC equipment to check the quality of the purified fructan mixture. We developed the miniaturized kinetic 1-FEH activity assay for application in routine physiological phenotyping, similar to the miniaturized carbohydrate enzyme activity assays (Jammer et al., 2015). Therefore, the entire procedure should be simple and fast, and available to many labs. Commercially available inulin from chicory root can be used as a substrate for 1-FEH activity measurements (Krivorotova and Sereikaite, 2014). However, in perennial ryegrass, 1-kestotriose is much more abundant (Pavis et al., 2001b) and, therefore, a more natural substrate for ryegrass 1-FEH than inulin. Comparison of both substrates indeed showed that the use of 1-kestotriose (**Figure 4** open circles), resulted in different 1-FEH activity at 1, 10, and 14 days of cold acclimation compared to inulin (*p* < 0.02; *t*-test; **Figure 4**, closed circles). Under control conditions both substrates resulted in comparable 1-FEH activities (**Figure 4**, *t* = 0). Lothier et al. (2007) demonstrated that glucose inhibits 1-FEH activity in perennial ryegrass. Considerable amounts of glucose were found in the commercial inulin preparation (data not shown). An inhibition of 1-FEH through glucose, can only partially explain why different results were obtained by inulin compared to 1-kestotriose as substrate, since differences in 1-FEH activity between 1-kestotriose and inulin were not detected for all time points. Interestingly, such differences in 1-FEH activity depending on the use of either inulin (Lothier et al., 2007) or 1,1-kestotetraose (Marx et al., 1997) as substrate have been found earlier. Similar, substrate specific results were obtained for a wheat 6&1-FEH (Kawakami et al., 2005). The presence of 1-FEH isoforms (Bonnett and Simpson, 1993, 1995; Marx et al., 1997; Lothier et al., 2007, 2014), preferentially degrading either low or high DP fructans and differently regulated during cold acclimation, might explain this substrate specific behavior. The sucrose produced using 1-kestotriose as substrate could be converted by invertase activity to fructose, leading to an overestimation of 1-FEH activity. However, the amount of sucrose generated by 1-FEH at the end of the enzyme coupled kinetic reaction is extremely low (18 μM) compared to the very high Km value of invertase for sucrose in the mM range and thus ca. two orders of magnitude higher. Further, the optimal pH for the 1-FEH reaction is 5.5 and for vacuolar invertase 4.5. In addition, invertase activities were shown to decrease during the cold treatment (**Figure 4**), while the increased 1-FEH activities correspond to the *1-FEH* expression values at days 10 and 14 (**Figure 4**). Together, the potential ‘contaminating’ fructose levels from background invertase activity would be indeed very low and thus negligible.

### Case Study: Effect of Cold Acclimation on 1-FEH Activity

Because 1-kestotriose is more abundant in perennial ryegrass (Pavis et al., 2001b) than high DP inulin, we considered the kinetic assays using this substrate to reflect the effect of cold acclimation on 1-FEH in our case study more precisely. 1-FEH activity showed an initial positive trend for the first 2 days of cold acclimation with significant change compared to control only at day 1 (*p* < 0.002; *t*-test), reaching control level again at day 3 (**Figure 4**). From days 4 to 14, 1-FEH activity increased continuously, however, only being significantly higher compared to the control (*t* = 0) at day 10 (*p* < 0.005; *t*-test) and 14 (*p* < 0.02; *t*-test). Cold induction of *FEH* genes has been observed in different species such as chicory (Kusch et al., 2009), wheat (Kawakami et al., 2005; Kawakami and Yoshida, 2012), *Bromus pictus* (del Viso et al., 2009) and *Poa pratensis* (Rao et al., 2011). In agreement with these inductions of *FEH* gene expression by cold, putative cold inducible ABRE and/or CRT/DRE elements have been identified in a promoter for chicory *1-FEHIIa* (Michiels et al., 2004). In perennial ryegrass, a corresponding heterologous cold inducible transcription factor (*LpCBF3*) was isolated (Xiong and Fei, 2006). In the mature leaves of *B. pictus*, the cold induction of *Bp1-FEHa* transcript was accompanied by an increase of 1-FEH activity (del Viso et al., 2009). This is in accordance with our findings of increased 1-FEH activity and *1-FEH* expression during cold acclimation. Fructans have been shown to insert into phospholipid membranes (Hincha et al., 2000; Vereyken et al., 2001; Livingston et al., 2009; Sandve et al., 2011) and thereby are supposed to protect plant cells from osmotic or frost damage. The role of increased FEH activities under osmotic stress conditions may be linked to the trimming function of FEHs (Bancal et al., 1992; Van den Ende et al., 2003; Lothier et al., 2007), providing low DP fructans for membrane stabilization.

### Integration of the Miniaturized Kinetic 1-FEH Assay in Physiological Phenotyping

The determination of the abundance of transcripts by microarray or RNAseq techniques is widely used as proxy to assess the function of enzymes involved in metabolic processes, the synthesis of metabolites or signaling molecules. Although such an approach has the potential to give valuable insights into regulatory networks, there is growing awareness that transcripts and protein abundance often poorly correlate (Stitt and Gibon, 2014). The correlations are even worse if it comes to enzyme activities that are, however, the main determinants of physiology. Based on this insight experimental platforms have been established to determine complex enzyme activity signatures in a microtiter plate scale for physiological phenotyping (Gibon et al., 2004; Sulpice et al., 2010; Jammer et al., 2015). Such an enzyme activity profile is a good approximation of the physiology of a cell and highly robust physiological marker (Biais et al., 2014) for physiological phenotyping within a multidimensional phenomics approach (Großkinsky et al., 2015). Since the currently established platforms to determine enzyme activity signatures lack any fructan active enzymes, the developed simple and fast enzyme coupled miniaturized kinetic 1-FEH enzyme activity assay is valuable to include also fructan metabolism as component of a physiological fingerprint (Großkinsky et al., 2015).

Important features of kinetic enzyme activity assay protocols are robustness of the protein extract preparation and assay conditions as well as wide applicability to diverse plant species and organs. This 1-FEH activity assay can be performed using the extraction buffer described by Pavis et al. (2001a) and subsequently following the same protein extraction procedure and workflow that is used to determine the activity profile of 13 key enzymes of primary carbohydrate metabolism (Jammer et al., 2015). Extensive optimizations were performed for the enzyme coupled kinetic 1-FEH assay to ensure that the influence from other enzymes in the same protein extract is minimal, evident from linearity in the activity plots. Based on our experience with the activity assays for enzyme of primary carbohydrate metabolism (Jammer et al., 2015), the reaction parameters are widely applicable to diverse organs and plant species and only in few specific cases required further optimization (fine tuning). Thus, our 1-FEH assay expands the available tool kit for physiological phenotyping to understand the dynamic temporal responses of fructan metabolism to external fluctuations and responses toward environmental conditions (Schurr et al., 2006). It contributes to closing the knowledge gap between phenotypes and their genetic bases and thus complements the earlier introduced analysis of hormone profiles (Großkinsky et al., 2014) and carbohydrate and nitrogen metabolism enzyme activity signatures (Sulpice et al., 2010; Jammer et al., 2015) to assess the complex genotype × environment × management interaction relevant for crop plant breeding (Großkinsky et al., 2015).

## Author Contributions

Designed the experiments: AG, AM-B, M-PP, EVDG, and TR. Performed the experiments: AG. Analyzed the data: AG, AM-B, and EVDG. Contributed reagents/materials/analysis tools: M-PP, EVDG, and TR. Wrote the paper: AG, AM-B, M-PP, EVDG, and TR.

## Conflict of Interest Statement

The authors declare that the research was conducted in the absence of any commercial or financial relationships that could be construed as a potential conflict of interest.

## Abbreviations

1-FEH: fructan 1-exohydrolase
6&1-FEH: fructan 6&1-exohydrolase
6-FEH: fructan 6-exohydrolase
ABRE: abscisic acid responsive element
ATP: adenosine triphosphate
*Bp1-FEHa*: *Bromus pictus 1-fructan exohydrolase a*
CRT/DRE: C repeat/dehydration responsive
DP: polymerization degree
HPLC: high performance liquid chromatography
*LpCBF3*: *Lolium perenne C-repeat binding factor*
NAD+: nicotinamide adenine dinucleotide(_ox,_)
NADH: nicotinamide adenine dinucleotide(_red,_)
*Pp-FEH*: *Poa pratensis fructan exohydrolase*
PVPP: polyvinylpyrrolidone
*Wfh-sm3*: *wheat fructan exohydrolase clone sm3*
